# Endogenous leptin promotes autophagy in EBSS-induced PFCs

**DOI:** 10.1080/19768354.2019.1651766

**Published:** 2019-08-16

**Authors:** Deling Jiao, Zhen Yang, Lulu Wang, Binyue Hu, Jing Wang, Anyong Xu, Wenmin Cheng, Baoyu Jia, Yubo Qing, Hong-Ye Zhao, Hong-Jiang Wei

**Affiliations:** a Key Laboratory of Animal Gene Editing and Animal Cloning in Yunnan Province, Kunming, People’s Republic of China; b Xenotransplantation Engineering Research Center in Yunnan Province, Kunming, People’s Republic of China; c State Key Laboratory for Conservation and Utilization of Bio-Resources in Yunnan, Yunnan Agricultural University, Kunming, People’s Republic of China; d College of Animal Science and Technology, Yunnan Agricultural University, Kunming, People’s Republic of China; e College of Veterinary Medicine, Yunnan Agricultural University, Kunming, People’s Republic of China

**Keywords:** Leptin, autophagy, PFCs, autophagy-related genes, autophagy signaling pathway-related genes

## Abstract

Leptin is an important adipokine and plays a vital role in animals. However, the role of leptin in the autophagic response of pig fibroblast cells (PFCs) has not been fully elucidated. In this study, we investigated the relationship between leptin and autophagy as well as underlying molecular basis. We found that PFCs treated with EBSS could secrete leptin, and the leptin concentration in the supernatant of leptin transgenic PFCs was higher than that of WT PFCs. We found an increase in LC3-II protein level and a decrease in p62 protein level in treated leptin transgenic PFCs compared with treated WT PFCs. Meanwhile, we observed an increase of autophagosomes by transmission electron microscopy and an enhancement of the accumulation of LC3 puncta in the cytoplasm of treated leptin transgenic PFCs, and these effects were further augmented by Baf A1 treatment. Furthermore, we detected the expression levels of 7 autophagy signaling pathway genes and 17 autophagy-related (ATG) genes by q-PCR. We found that between the two types of EBSS-treated cells 3 genes expression pattern were significantly different among the 7 autophagy signaling pathway genes and 8 genes expression pattern were significantly differernt among the ATG genes. These results indicated that leptin may promote autophagy and involving the downregulation of FOXO1 and LMNA genes via an unknown pathway which causes the upregulation of the 4 genes and the downregulation of 4 genes.

## 1. Introduction

Leptin is a polypeptide hormone secreted mainly by animal adipocytes and encoded by the OB gene (Matarese et al. 2010). As we know leptin can be secreted by non-adipose tissue, including placenta, human gastric mucosa, hepatic stellate cells, mammary epithelial cells, pituitary gland, brain and skeletal muscle (Himms-Hagen 1999). Moreover, leptin is involved in signaling pathways regulating cell proliferation, differentiation and survival (Villanueva and Myers 2008), autophagy (Cassano et al. 2014) and nutrient metabolism (Varela and Horvath 2012).

Autophagy is a cellular metabolic process that maintains physiological function and homeostasis through liposomal protein degradation. Autophagy plays an important role in the growth and development of animals, cell differentiation and the occurrence and development of diseases (Gozuacik and Kimchi 2004). In addition, abnormal autophagy can lead to many diseases, such as energy metabolis (Jacob et al. 2017) and immune-related diseases (Deretic et al. 2013).

Previous studies had reported that leptin can modulate autophagy. In human intervertebral discs, leptin can promote autophagy of degenerative nucleus pulposus cells by promoting the expression of phosphorylated AKT protein and activating the ERK-mTOR signaling pathway (Zhang et al. 2018). However, in the lung tissue leptin promotes pulmonary fibrosis development by inhibiting autophagy via PI3 K/Akt/mTOR pathway (Gui et al. 2018). The role of leptin in autophagy is argumentative, may be leptin regulate autophagy in a tissue-specific manner (Piekarski et al. 2018). The role of leptin in the autophagic response of PFCs has not been fully elucidated. In this study we established a new biological model to support further study of the molecular mechanism of leptin in modulating autophagy in response to starvation.

## 2. Materials and methods

### 2.1. Reagents and antibodies

EBSS were purchased from Gibco (24010-043, USA). Antibodies against LC3B and p62/SQSTM1 were obtained from Sigma-Aldrich (L7543 and P0067), and β-actin antibody was purchased from Sigma-Aldrich (A5441). Bafilomycin (Baf A1) was purchased from sangon biotech (shanghai, China)

### 2.2. Cell culture

The WT and leptin transgenic PFCs were isolated and established from ear tissues of WT and leptin transgenic pigs (Chen et al. 2018). The WT and leptin transgenic PFCs were cultured in basic (1×) Dulbecco’s modified Eagle’s medium (DMEM) (Gibco, NY, USA) supplemented with 10% fetal bovine serum (Ausbian, VS500 T) and 100 IU/ml penicillin. The cells were seeded in gelatin-coated 10 cm dishes and cultured in 10 ml of medium at 37°C.

### 2.3 . ELISAs for leptin concentration in culture supernatant

The leptin level in the cell culture supernatant was detected by an ELISA kit (Meilian Biology, ml002355, Shanghai, China). Cell culture supernatant was collected by sterile centrifugal tubes and centrifuged for 20 min at 2000rpm. Standard wells were added to 50 μl of different concentrations of standard reagents, and 40 μl of sample dilution reagent and 10 μl of cell culture supernatant were added to the sample wells. Then, 100 µl of enzyme labeling reagent was added to each well except the blank well, and the sample was incubated at 37°C for 60 min. Then, the enzyme-labeled plate was washed with washing solution five times for 30 s each time. First, 50 µl of developer A was added to each well, followed by 50 µl of the developer B, and the samples were incubated at 37°C for 15 min. The absorbance of each well was measured by zero-setting the blank well at an Enzyme – labeled meter (Thermo scientific, MULTISKAN GO) in 450 nm.

### 2.4. Quantitative real-time polymerase chain reaction (q-PCR) assays

WT and leptin transgenic PFCs were treated with EBSS for 4 h. Total RNA was isolated using TRIpure reagent (BioTeke, China) according to the manufacturer’s instructions. cDNA was synthesized from total RNA using a PrimeScript RT reagent kit (TaKaRa, Japan). The obtained cDNA was used as a template in SYBR Green-based q-PCR (CFX-96, Bio-Rad, Hercules, CA, USA). The mRNA levels of the ATG and autophagy signaling pathway-related genes were assessed with quantitative polymerase chain reaction (q-PCR). GAPDH was used for normalization. Most primer sequences were the same as our previous study which will soon be published in Translational Cancer Research (Fei et al. 2019), and additional primer sequences are listed in Table S1.

### 2.5. Protein extraction and immunoblotting

WT and leptin transgenic PFCs were treated with EBSS for 0 and 4 h after pretreatment with 100 nM Baf A1 for 2 h. Cells were washed twice with phosphate buffered saline (PBS) and collected. Then, the total protein concentration of cell lysates was determined using a BCA protein assay kit (Beyotime, Shanghai, China). Protein samples (total protein: 20 μg) were separated by 12% sodium dodecyl sulfate-polyacrylamide gel electrophoresis and transferred onto a polyvinylidene fluoride membrane. The membranes were incubated for 60 min in 5% bovine serum albumin (BSA) buffer (Solarbio, Beijing, China) with gentle shaking to block nonspecific binding before incubation with the diluted primary antibody LC3-II (rabbit, 1:1000) and p62 (rabbit, 1:1000) overnight at 4°C. Subsequently, the membranes were incubated with the secondary antibody (goat anti-rabbit, 1:5000, Santa Cruz, CA, USA) for 90 min at room temperature. The membrane was washed three times in PBS for 10 min each time, and the membrane was incubated for 3 min with a chemiluminescence (ECL) reagent (Easy See Western blot Kit, Transgene, Alsace, France). Finally, the membranes were exposed with an imaging system (Bio-Rad, Hercules, USA).

### 2.6. Transmission electron microscopy

WT and leptin transgenic PFCs were treated with EBSS for 4 h and were fixed overnight at 4°C using 2.5% glutaraldehyde in PBS. Afterward, samples were postfixed with 1% OsO_4_ for 2 h at 4°C, followed by serial ethanol dehydration and embedding in Epon 812 resin. Serial sections of uniform thicknesses of approximately 60 nm were made using a Leica UC7 ultramicrotome. After staining with 2% uranyl acetate and lead citrate, ultrathin sections were examined using a transmission electron microscope (JEM 1400 plus, JEOL, Japan).

### 2.7. Confocal microscopy

WT and leptin transgenic PFCs were treated with EBSS for 0 and 4 h after pretreatment with 100 nM Baf A1 for 2 h. The cells were seeded onto 6-well culture slides and treated with EBSS for 4 h, which were fixed with formaldehyde for 10 min and then blocked with a buffer containing 5.0% BSA and 0.5% Triton X-100 for 30 min. Next, the cells were incubated with a primary antibody against LC3-II (1:200) at 4°C overnight. Then, the cells were incubated for 1 h with secondary FITC-conjugated antibody (1:400) to visualize the binding sites of the primary antibody with laser confocal microscopy (OLYMPUS FV1000, Tokyo, Japan).

### 2.8. Statistical analysis

Statistical comparisons were performed using Student’s *t*-test. Quantitative data are expressed as the mean ± SD. *p < 0.05 and **p < 0.01 versus the control were considered significant.

## 3. Results

### 3.1. Starvation induces autophagy in WT and leptin transgenic PFCs

**Figure 1.**
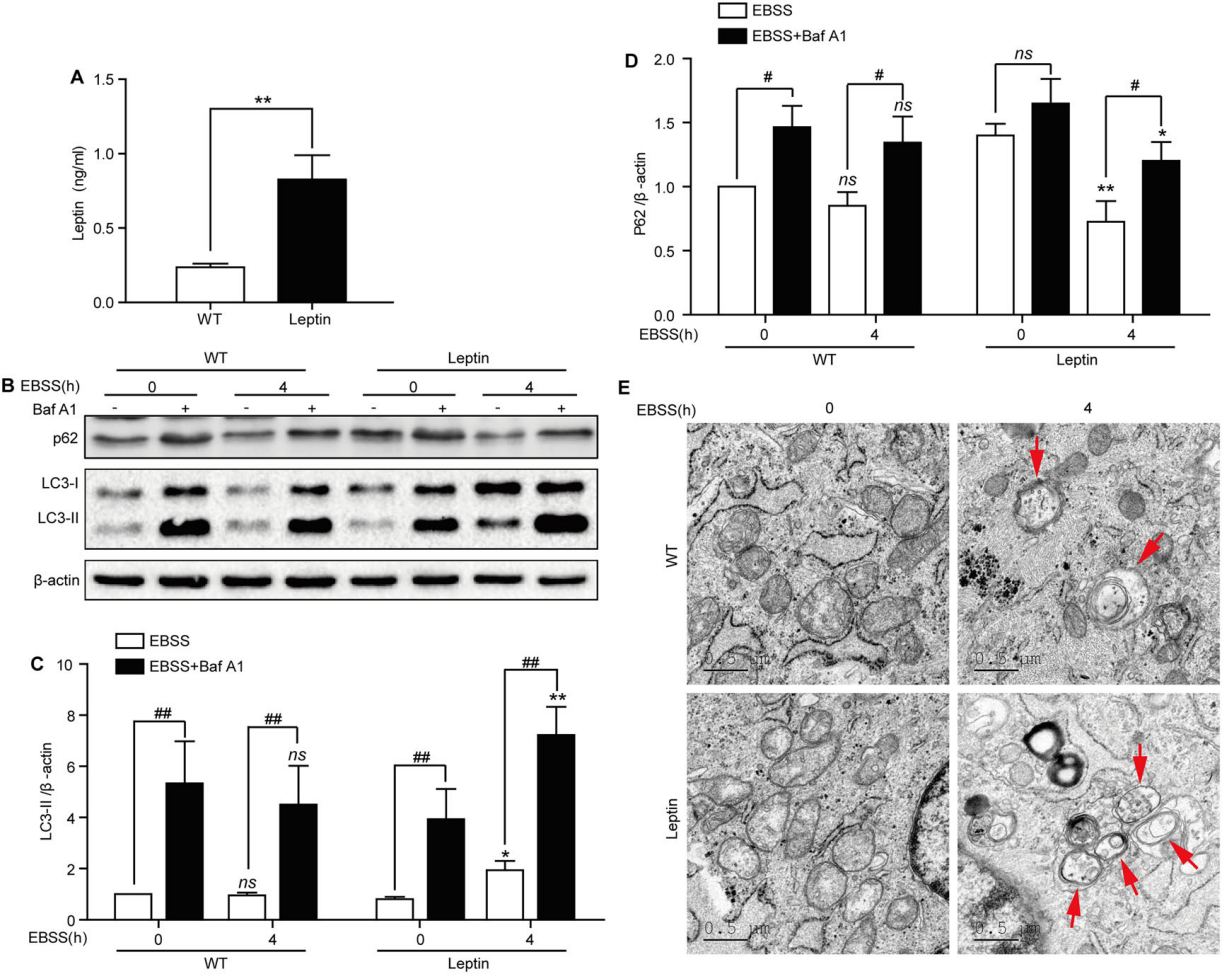
Starvation induces autophagy in WT and leptin transgenic PFCs. (A) Quantification of the leptin concentration in culture supernatants of WT and leptin transgenic PFCs. ** *p* < 0.01 compared to WT PFCs. (B) Both PFCs were treated with EBSS for the indicated times in the presence or absence of Baf A1 (100 nM); then, the protein expression levels of LC3B and p62 were analyzed by immunoblotting. *β*-actin was used as an internal control. (C-D) Quantification of the LC3-II and P62 protein expression level in both treated cells. *p < 0.05 and **p < 0.01 compared to EBSS-treated PFCs or EBSS with Baf A1-treated PFCs at 0 h, ^#^*p* < 0.05 and ^##^ *p* < 0.01 compared to EBSS-treated PFCs at the indicated time. (E) Representative electron micrographs for both cells. WT and leptin transgenic PFCs were treated with EBSS for 4 h. Red arrows refer to autophagic vacuoles. Scale bars = 0.5 μm.

The leptin protein is secreted from cells, especially from adipocytes. To determine whether leptin was also secreted from PFCs, we detected the leptin concentration in the culture supernatant. The ELISA results showed that the leptin concentration was significantly increased in leptin transgenic PFCs compared with WT PFCs (Figure 1(A)). Thus, we further investigated the role of leptin on autophagy in both types of PFCs.

The most widely used autophagic flux detection method is analysis of LC3-II protein level in the presence or absence of Baf A1, which is a lysosomal inhibitor (Noboru and Tamotsu 2007; Mizushima et al. 2010). We found that the expression level of P62 decreased and the expression level of LC3-II increased after EBSS treatment at 4 h in leptin transgenic PFCs, while the expression of LC3-II and P62 was not changed in WT PFCs. The expression level of LC3-II and P62 was further enhanced after combination treatment with EBSS and Baf A1 (Figure 1(B–D)). Meanwhile, we observed that the number of autophagosomes increased in treated leptin transgenic PFCs compared with treated WT PFCs by transmission electron microscopy (Figure 1(E)).

**Figure 2.**
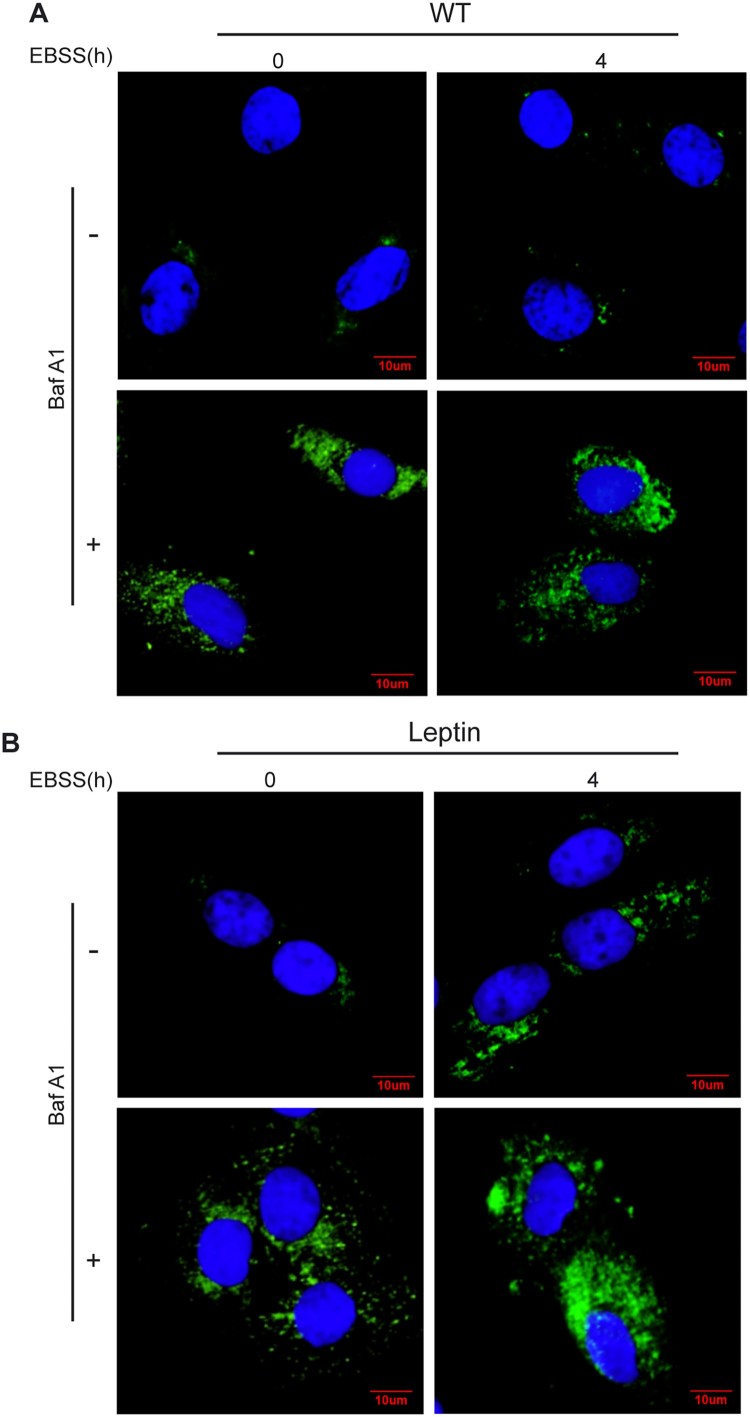
Confocal microscopy images of LC3 in WT and leptin transgenic PFCs. Both leptin transgenic and WT PFCs were treated with EBSS or a combination of EBSS with Baf A1 for the indicated times, and the accumulation of LC3 puncta was analyzed with confocal microscopy. (A) Representative images of LC3 puncta in WT PFCs. (B) Representative images of LC3 puncta in leptin transgenic PFCs. Cells were stained with antibodies against LC3 (green), and nuclei were stained blue with DAPI. Scale bars = 20 μm.

To further determine whether EBSS-induced starvation can induce autophagy in WT and leptin transgenic PFCs, both cells were treated by EBSS for 4 h with or without Baf A1. The confocal microscopy images showed that the LC3 puncta in the cytoplasm was increased after treatment with EBSS or a combination with EBSS and Baf A1for 4 h in leptin transgenic PFCs, while the number of LC3 puncta showed no change in WT PFCs (Figure 2). These results demonstrated that starvation induced autophagy in leptin transgenic PFCs at 4 h.

### 3.2. Effect of leptin on the mRNA level of autophagy signaling pathway-related genes in PFCs

**Figure 3.**
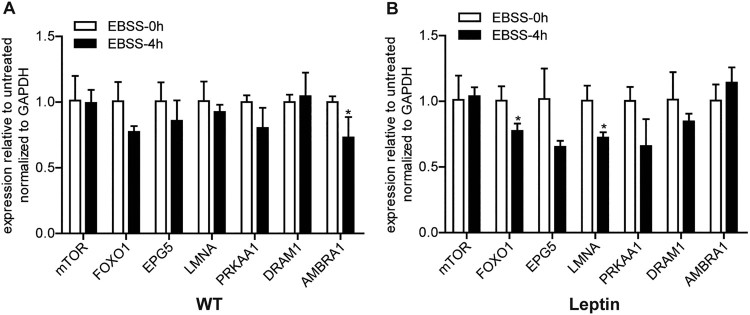
Effect of leptin on the mRNA expression levels of autophagy signaling pathway-related genes in PFCs. Both types of cells were treated with EBSS for 0 and 4 h. (A, B) The mRNA expression levels of the autophagy signaling pathway-related genes mTOR, FOXO1, EPG5, LMNA, PRKAA1, DRAM1 and AMBRA in both treated cells. *p < 0.05 compared to the EBSS treatment at 0 h in both PFCs.

Some studies have shown that the activation of autophagy is regulated by autophagy-related signaling pathways (Ruderman et al. 2010). As shown in Figure 3, there was no significant difference in the mRNA levels of mTOR, EPG5, PRKAA1 and DRAM1 between WT and leptin transgenic PFCs treated with EBSS for 4 h (Figure 3(A, B)). Compared with the 0 h group, AMBRA1 was significantly downregulated in EBSS-treated WT PFCs (Figure 3(A)) but not in the treated leptin transgenic PFCs. In the treated leptin transgenic PFCs, the expression level of FOXO1 and LMNA mRNA was significantly downregulated (Figure 3(A, B)) but there was no change in the EBSS-treated 4 h WT PFCs. These results demonstrated that the partial autophagy signaling pathway response might be different in the two types of PFCs.

### 3.3. Effect of leptin on mRNA expression levels of ATG genes in PFCs

**Figure 4.**
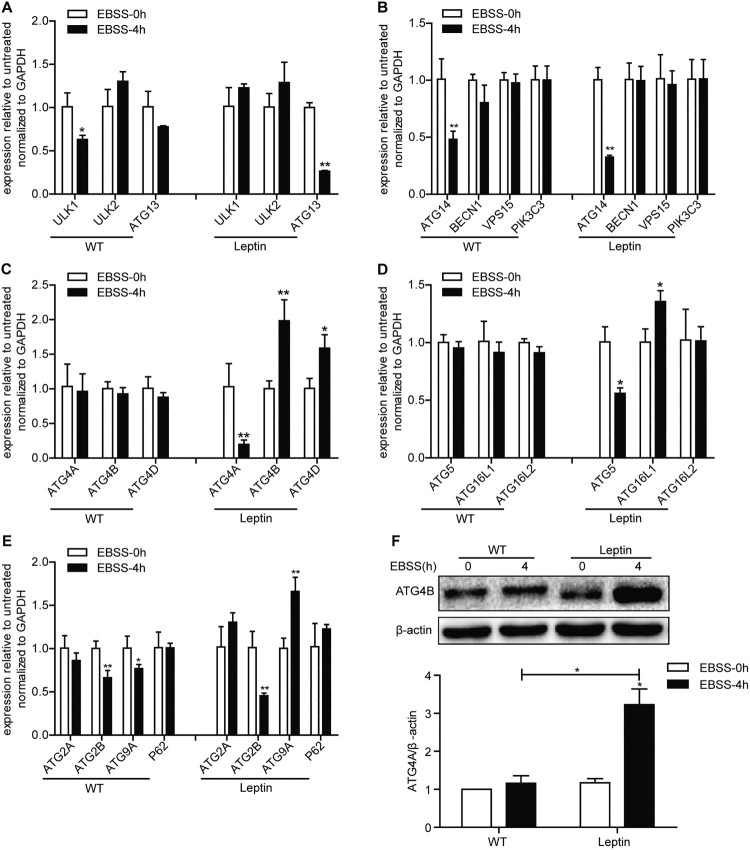
Effects of leptin on the mRNA level of ATG genes in PFCs. The cells were treated as described above. (A) The mRNA expression levels of the autophagosome initiation-related genes ULK1, ULK2 and ATG13 in both treated cell. (B) The mRNA expression levels of the nucleation-related genes ATG14, BECN1, VPS15 and PIK3C3 in both treated cells. (C) The mRNA expression levels of the elongation-related genes ATG4A, ATG4B and ATG4D in both treated cells. (D) The mRNA expression levels of the elongation-related genes ATG5, ATG16L1 and ATG16L2 in both treated cells. (E) The mRNA expression levels of the degradation cycling-related genes ATG2A, ATG2B, ATG9A and P62 in both treated cells. (F) The protein level of ATG4B in both treated cells was analyzed by immunoblotting. β-actin was used as an internal control. *p < 0.05 and **p < 0.01 compared to the EBSS treatment at 0 h in both PFCs.

The ATGs are involved in the formation of autophagosomes. The results showed that the mRNA levels of the ATG14 and ATG2B genes in WT and leptin transgenic PFCs were significantly down-regulated by starvation, but there was no change in the mRNA expression levels of the ULK2, BECN1, VPS15, PIK3C3, ATG16L2, ATG2A and P62 genes (Figure 4(A, B, D, E)). In the EBSS-treated 4 h WT PFCs, the mRNA levels of the ULK1 and ATG9A genes were significantly downregulated compared with the WT 0 h group. However, in the EBSS-treated leptin transgenic PFCs, the mRNA levels of the ULK1 gene did not change and ATG9A increased (Figure 4(A, E)). The mRNA levels of ATG13, ATG5 and ATG4A decreased significantly in the EBSS-treated leptin transgenic PFCs, but there was no change in the EBSS-treated WT PFCs (Figure 4(A, C, D)). The expression levels of the ATG4B, ATG4D, ATG16L1 genes were significantly upregulated in the EBSS-treated leptin transgenic PFCs, while the mRNA levels of ATG4B, ATG4D and ATG16L1 did not change and ATG9A decreased significantly in the EBSS-treated WT PFCs (Figure 4(C, D, E)). Additionally, the protein level of ATG4B increased in the EBSS-treated leptin transgenic PFCs compared with the EBSS-treated WT PFCs at 4 h (Figure 4(F)). These results demonstrated that the distinctly differences in both types of EBSS-treated PFCs.

## 4. Discussion

Autophagy is involved in a wide range of physiological and pathological processes of various diseases (Levine and Kroemer 2008). Leptin has been reported to regulate autophagy and plays a critical role in this process (Gui et al. 2018; Zhang et al. 2018). Fibroblast cells and adipose cells share a common stem cell origin. A. Glasow detected and reported that human fibroblast cells could secrete leptin (Glasow et al. 2001). In our study, we also found that leptin was present in the culture supernatant of WT and leptin transgenic PFCs, which was consistent with Glasow’s research. Moreover, the concentration of leptin was higher in leptin transgenic PFCs than in WT PFCs (Figure 1(A)).

Leptin has long been recognized to play an important role in the regulation of autophagy. Leptin can inhibit (Luo et al. 2016) or promote (Nepal et al. 2015) autophagy, due to the different types of tissue. In breast cancer cells, leptin promoted growth of breast cancer cells via autophagy activation (Raut et al. 2017). Leptin promotes autophagy of mesenchymal stem cells and protects them from apoptosis (Wang et al. 2014). In this study, leptin also showed the promoting effects on autophagy in the leptin transgenic PFCs, which were consistent with previous studies. So we showed that leptin can promote autophagy in PFCs.

Autophagy is also regulated by autophagy-related upstream signaling pathway genes. The AMPK/mTOR, PI3K/mTOR and PI3K/Akt signaling pathways are common signaling pathways between the leptin and autophagy signaling pathways, which play an important role in autophagy (Ruderman et al. 2010). Leptin treatment can inhibit the activation of mTOR in the progression of pulmonary fibrosis (Gui et al. 2018), and mTOR is an important inhibitor of autophagy upstream (Jung et al. 2009). In this study, the expression level of mTOR mRNA did not change significantly. Leptin may promote autophagy by affecting the expression of phosphorylated mTOR protein, but there was no effect on total mTOR protein and mRNA. It has been reported that LMNA^−/−^ mice attributed to elevated MTORC1 signaling leading to impairment of autophagic flux (Ramos et al. 2013). Moreover, in LMNA mutation mice the improved heart function associated with pharmacological blockade of MTOR was correlated with enhanced autophagy (Choi and Worman 2013). Thus, the deficiency of LMNA may promote the occurrence of autophagy. In our study, the mRNA level of LMNA decreased in the treated leptin transgenic PFCs but not changed in the treated WT PFCs. However, autophagy was actived in the treated leptin transgenic PFCs. In PFCs leptin may promote autophagy via inhibiting the expression of LMNA. In this study, FOXO1 decreased significantly in the treated leptin transgenic PFCs but not in the treated WT PFCs (Figure 3(A, B)). FOXO1 was shown to mediate autophagy (Wang et al. 2016), but in this study, FOXO1 decreased in the treated leptin transgenic PFCs, which is not consistent with the previous reports. Thus, the functions of mTOR, LMNA and FOXO1 on Leptin promoted autophagy in PFCs require further study.

In addition, the effects of leptin on the ATG genes were notable. The mRNA levels of ATG13, ATG5 and ATG4A decreased significantly in the treated leptin transgenic PFCs, but did not changed in the treated WT PFCs (Figure 4(A, C, D)). It has been reported that the interactions of ATG13 with protein and lipids for potential modulation of ULK1 complex formation and autophagy induction (Wallot-Hieke et al. 2018). ATG5 was thought to induce autophagy via PERK signaling (Zheng et al. 2019), but in this study ATG5 decreased in the treated leptin transgenic PFCs, the function of ATG5 in the leptin transgenic PFCs still need further study. Our results showed that the expression levels of ATG4B, ATG4D, ATG16L1 and ATG9A were upregulated in the treated leptin transgenic PFCs but not in the treated WT PFCs (Figure 4(C, D, E)). ATG4B was reported to mediate the cleavage of Pro-LC3 to promote the binding of LC3 to lipids in autophagosomes. The phosphorylation-deficient ATG4B cells showed an impaired autophagic flux (Yang et al. 2015). The protein level of ATG4B matched the mRNA level in the protein validation experiment (Figure 4(F)). Therefore, the increase in ATG4B could be one of the manifestations of autophagic enhancement. This finding suggests that leptin may enhance autophagy by upregulating ATG4B. ATG9A is a multispanning membrane protein essential for autophagy. It has been reported that ATG9A could shape the forming autophagosome to promote autophagy (Judith et al. 2019). Thus, leptin may promote autophagy by increasing ATG9A. ATG16L1 is a core autophagy protein implicated at distinct phases of autophagosome biogenesis. Study has reported that regulated recruitment of ATG16L1 to the pre-autophagosomal structure is required for its autophagic activity (Dudley et al. 2019). As ATG16L1 increased in the treated leptin transgenic PFCs, so leptin may promote autophagy by upregulating expression of ATG16L1.

In summary, our findings indicated that leptin positively regulated autophagy via unknown pathways to downregulate the expression level of FOXO1 and LMNA genes, which causes the upregulation of the LC3, ATG4B, ATG16L1 and ATG9A genes and downregulation of the ATG13, ATG4A, ATG5 and P62 genes. These findings will provide a new biological model and support further study of the molecular mechanism of leptin in controlling autophagy in response to starvation.

## Supplementary Material

Supplemental Material
