# Supplementary material for: Endogenous leptin promotes autophagy in EBSS-induced PFCs
Source: Anim Cells Syst (Seoul). 2019 Aug 16;23(5):318–25. doi: 10.1080/19768354.2019.1651766 (PMC6830286; doi:10.1080/19768354.2019.1651766)
Supplement: Supplemental Material [file TACS_A_1651766_SM4686.docx]

Table S1. The partial primer sequences for qPCR

| Gene | Primers sequence (5’ to 3’) | |
| --- | --- | --- |
| ATG13  ATG14 | F: GTGGAGGCAGGAGTAGGATGG  F: GAAAGCACTACGCTCTAGGTTATGT | R：TGGGAATGGCTGACTTCAGAGT  R：GAAGGACCGTGACCCTCTGTT |
| ATG4D | F: CTCTACCTGGACCCTCACTACTGCC | R: GAGCGTCTCAAACTCCTTCCTGTCT |
| ATG5 | F: TGAGCGAGCATCTGAGCTATCC | R: GCCTCCACCAAACCTGACTGA |
| ATG16L2 | F: CCCTCTAGTCGAAGCCTGAAGCC | R: TGAAACAATACCGTAATCCGAACCC |
| PRKAA1 | F: TCCATATTATTTGCGTGTTCG | R: CTCTGTGGAGTAGCAGTCCCT |
